# Microbiota-mediated blocking of *plasmodium falciparum* sporozoites in *Anopheles*: a systematic review

**DOI:** 10.3389/finsc.2026.1958166

**Published:** 2026-10-26

**Authors:** Celestina O. Olafusi, Mercy E. Tebamifor, Tobiloba I. Bejapade, Israel S. Afolabi, Olubanke O. Ogunlana

**Affiliations:** 1 Department of Biochemistry, Covenant University, Ota, Ogun State, Nigeria; 2 Covenant Applied Informatics and Communication Africa Centre of Excellence, Covenant University, Ota, Ogun State, Nigeria; 3 Department of Biochemistry, University of Medical Sciences, Ondo, Ondo State, Nigeria

**Keywords:** *Anopheles* mosquitoes, microbiome, paratransgenesis, *Plasmodium falciparum*, salivary gland, sporozoite

## Abstract

**Introduction:**

The development of *Plasmodium falciparum* sporozoites in the mosquito, *Anopheles*, is essential for malaria transmission. Despite the unsurprising fact that the mosquito microbiota has become a major factor in the modulation of the competence of mosquitoes, the specific types of microbial taxa and mechanisms affecting the development of the sporozoite are not well synthesized. This systematic review brings together and critically evaluates the available evidence to understand the role of microbiota in the development of *P. falciparum* sporozoites in *Anopheles* mosquitoes with a specific focus on the salivary gland microbiome.

**Methods:**

A systematic search of PubMed, Scopus, and Google Scholar databases (2000-2026) was conducted, and 41 studies met the inclusion criteria. Extraction and synthesis of data were conducted in the form of a narrative in accordance with the PRISMA guidelines. Risk of bias was assessed using SYRCLE’s tool for animal studies.

**Results:**

The *Wolbachia* effects varied by strain and species: wAlbB caused a 3.4- to 3.7-fold decrease in sporozoites, wAnga-Mali had a negative correlation with 95% presence, and there was no correlation for wAnmo in *An. moucheti*. All African countries (1–57% prevalence) were consistently affected by *Microsporidia MB*, with no fitness costs. *Serratia ureilytica* Su_YN1 inhibited ~98% of parasites using outer membrane vesicles loaded with AmLip, which targets parasites via the phosphatidylcholine scavenging pathway. 93% transmission blocking was obtained in mouse models with engineered symbionts (transgenesis + paratransgenesis).

**Discussion:**

The mosquito microbiota plays a significant, but strain-, species-, and context-dependent role in the development of *P. falciparum* sporozoites. The *Microsporidia* and *Serratia* species are promising candidates for field deployment, offering sustainable, environmentally friendly complements to chemical insecticides for malaria vector control.

## 1 Introduction

Malaria is a significant global health problem, and in 2024 there are estimated to be 282 million malaria cases reported, mainly in sub-Saharan Africa in children under 5 years of age (1). Although vector control using insecticide-treated nets and artemisinin combination therapies has made substantial progress, gains have stalled due to the emergence and spread of insecticide resistance and parasite antimalarial resistance (2, 3). The challenges faced highlight the critical need for complementary and new approaches for interrupting malaria transmission. Humans are only capable of contracting *P. falciparum* when the mosquito vector, mainly *Anopheles gambiae*, *Anopheles coluzzii*, *Anopheles arabiensis*, and *Anopheles funestus* in sub-Saharan Africa, completes a complex developmental process (4, 5). The mosquito infects a human host and consumes gametocytes, the sexual stage of the parasite; gametocytes in the mosquito midgut get fertilized by the other gametocyte to form zygotes, which differentiate into mature motile ookinetes (6). These ookinetes pass through the midgut epithelium and grow to form oocysts on the basal side of the midgut. They start to multiply in a multiplicative fashion, and each oocyst forms thousands of sporozoites in the haemocoel. The sporozoites then move to and invade the salivary glands, stored there until they are transmitted to another human host during a subsequent blood meal (7). This developmental pathway is a time of extreme bottleneck, particularly between ookinete and oocyst and when sporozoites are invading the salivary glands, and is an appealing target for transmission-blocking interventions (TBIs) (8). Mosquito microbiota consists of bacteria, fungi, and microsporidia (9–11) that are acquired from the environment during their development, sugar feeding, and blood meal, and are retained within their life stages (12–14). Mounting evidence indicates that this microbial community actively modulates mosquito physiology, immunity, and vector competence for *Plasmodium* infection (15, 16). Recent syntheses have highlighted the gut microbiome’s potential role in determining susceptibility to malaria and emerging opportunities for microbiota-based transmission-blocking interventions (17). The effect of the midgut microbiota on *Plasmodium* infection has been studied in detail. Antibiotic treatment eliminating the microbiota has been shown to render mosquitoes more susceptible to infection with *P. falciparum*, thus highlighting the importance of microbiota in the mosquito’s natural refractoriness to infection (18–20).

On the other hand, specific bacterial species can be reintroduced or enriched to inhibit malaria parasite development in mosquitoes (16, 17, 21–25). Mechanistically, the microbiota can affect *Plasmodium* development in several ways: (1) direct inhibition via production of anti-parasitic factors such as ROS or secreted enzymes (26); (2) indirect effects by activation of the mosquito innate immune system, specifically the Immune Deficiency (IMD) pathway, which generates anti-parasitic effectors that also cross-react with parasites (27); and (3) modulation of the physical barrier function of the midgut, such as peritrophic matrix (28). The role of these mechanisms in different bacterial taxa is variable and likely influenced by the composition of the bacterial microbiota, which may be highly variable among mosquito species and populations and with environmental conditions (29).

The most promising bacterial candidates for paratransgenic or symbiont-based malaria control are members of the genera *Serratia*, *Enterobacter*, *Pantoea*, *Asaia*, the endosymbiotic bacterium *Wolbachia*, and the microsporidian fungus *Microsporidia MB* (30–32). The *Serratia ureilytica* strain Su_YN1 isolated from wild *Anopheles sinensis* in China has been found to inhibit the development of *P. falciparum* by secreting outer membrane vesicles (OMVs) containing the antimalarial lipase AmLip, which is delivered to the parasites (33). More recently, *Serratia* strains have been isolated from field-caught mosquitoes in Burkina Faso, and molecular analyses have confirmed their presence and possible use as *Plasmodium*-blocking agents (34). *Enterobacter* sp. Esp_Z, isolated from wild Zambian mosquitoes, produces ROS in a dose-dependent manner that is independent of the mosquito’s IMD immune pathway (21). Anti-plasmodial effectors from engineered *Pantoea agglomerans* cultured with oocysts can inhibit up to 98% of oocyst formation (35). *Microsporidia MB* is a vertically transmitted microsporidian symbiont found in the salivary glands of *An. arabiensis* and other vectors and has been demonstrated to reduce transmission of *P. falciparum* in the mosquito by preventing sporozoite colonization of the salivary glands at no apparent cost to the mosquito (36). Recent analyses have also provided more insight into the life cycle of *Microsporidia MB*, which has been shown to infect germline tissues of the mosquito and is capable of vertical and horizontal transmission (37, 38). These results highlight the potential of microbiota-based strategies as sustainable alternatives to chemical insecticides. However, temperature is a critical environmental determinant of *Microsporidia MB* dissemination potential that must be considered for field applications. A recent controlled laboratory study demonstrated that rearing *An. arabiensis* at 32 °C significantly enhanced larval development and *Microsporidia MB* infection rates, increasing dissemination potential 4.7-fold compared with 22 °C and 1.7-fold compared with 37 °C, despite approximately 20% higher mortality than at 27 °C. Lower temperatures (22 °C) yielded the lowest symbiont prevalence, suggesting that loss during vertical transmission is more likely under cooler conditions. Rearing temperatures between 27 °C and 32 °C, particularly closer to 32 °C, therefore appear optimal for maintaining high symbiont intensity and supporting rapid population growth of infected mosquitoes, with direct implications for the geographical deployment of *Microsporidia MB*-based interventions (39).

Of particular interest is *Wolbachia*, an intracellular, endosymbiotic bacterium, which has been suggested to modify insect reproduction and to inhibit pathogens in insects, a feature that has made it an exciting approach to malaria control (40). While *Anopheles* mosquitoes were previously considered naturally free of *Wolbachia*, there are now reports of natural infections in several populations in Africa, such as *An. gambiae*, *An. coluzzii*, *An. arabiensis*, *An. funestus* and *An. moucheti* (41–44). The impact of *Wolbachia* on *Plasmodium* development is strain- and species-specific, however. For example, the *wAlbB* strain with stable transinfection in *An. stephensi* significantly suppresses the oocyst and sporozoite load via ROS-mediated mechanisms (45). Transcriptomic analyses have identified immune genes that are responsive to *Wolbachia* infection, including TEP4, TEP15, lysozyme C2, CLIPB2, CLIPB4, and PGRP-LD, which are associated with *Wolbachia* suppression of *P. falciparum* infection (46). In contrast, the naturally occurring *Wolbachia* in *An. moucheti* does not correlate with the prevalence of *P. falciparum* infection, despite near-fixation prevalence (47). A recent study in Tanzania has been conducted to report, for the first time, the presence of supergroup B *Wolbachia* strains within wild populations of *An. funestus* s.l. At high prevalence (up to 70.8%), surprisingly low frequencies of co-infections with *P. falciparum* were detected in these strains using nested PCR (48). The contrasting results suggest the complexity of host-microbe-parasite interactions and the necessity of systematic assessment of these associations among vector species, geographical regions, and ecological conditions.

The microbiota of the midgut has been studied to a great extent, but the microbiota of the salivary glands, where the sporozoites develop and await transmission, has been under-studied. In fact, the final and most important anatomical structure for parasite transmission is the salivary gland, where the sporozoites are required to invade and establish infection to render them infectious to humans (49). In recent years, the salivary gland microbiome of wild *Anopheles* has started to be characterized (14, 50, 51), and studies have begun to explore the possible function of the salivary gland microbiome in the modulation of sporozoite development. Specific bacteria or microsporidia in salivary glands can affect sporozoite survival, infectivity, or potential for transmission directly, through immune priming, nutrient competition, anti-parasitic products, or other factors. Therefore, the composition and function of the salivary gland microbiome are crucial factors in discovering new targets for transmission-blocking interventions. In recent studies, it has been shown that mosquito saliva is a vector of bacteria that may be transferred to the mammalian host during blood feeding, posing important questions about the role of the salivary gland microbiome in pathogen transmission dynamics (14).

Most importantly, this review focuses on the salivary gland microbiome, but the data collected are, to a large extent, indirect. The majority of studies included measured sporozoite load in the salivary glands as an endpoint after manipulating microbiota in the midgut, instead of quantifying the actual salivary gland microbial communities. Little work has been done studying the microbial community of the salivary gland in mice, and only a few mechanistic studies have explored the effects of microbes residing in the salivary gland on the development of sporozoites. This is further discussed in the discussion section. To clarify the rationale for the salivary-gland emphasis in this review: while microbiota manipulation is primarily performed in the midgut, the salivary gland is the outcome site, the compartment where sporozoites must accumulate to render a mosquito infectious. Salivary gland sporozoite load is therefore treated as the primary endpoint, even when interventions are applied elsewhere. Direct characterization of the salivary gland microbiome nevertheless remains a major unmet research need, reflected in the small number of eligible studies that profiled this tissue. Despite growing interest in mosquito microbiota-*Plasmodium* interactions, critical knowledge gaps persist. First, the composition and functional role of the salivary gland microbiome, the anatomical site of sporozoite transmission, remains underexplored compared to the midgut microbiota. Second, the specific microbial taxa and molecular mechanisms best suited to inhibit sporozoite maturation and transmission are not systematically compared across studies, limiting the ability to identify leading candidates for field deployment. Third, context-dependent effects (variation by vector species, parasite strain, and ecological niche) reduce generalizability of single-study findings, yet no comprehensive synthesis has synthesized these associations. To date, there has been no systematic review that evaluates the totality of evidence linking mosquito microbiota to *Plasmodium falciparum* sporozoite development across laboratory and field studies.

The main aim of this systematic review is to gather and critically assess the existing knowledge regarding the role of the microbiota in the development of sporozoite *P. falciparum* in *Anopheles* mosquitoes, especially the salivary gland microbiome. Specifically, this review addresses the following questions: (1) What bacterial taxa or microbial communities are identified in the salivary glands and/or midgut of *Anopheles* mosquitoes? (2) How do specific microbial genera or species correlate with increased or decreased *P. falciparum* sporozoite development or transmission potential? (3) What molecular or biochemical mechanisms are proposed to explain these interactions? (4) What methods are used to manipulate or assess microbiota in mosquitoes? (5) Are any microbial candidates identified that could be leveraged for paratransgenic strategies to inhibit sporozoite maturation or transmission? By synthesizing evidence from laboratory and field studies, this review aims to identify microbial influences and possible candidates for vector-based malaria control strategies, ultimately contributing to the development of novel, sustainable interventions to combat malaria.

## 2 Method

This systematic review was carried out to uphold the Preferred Reporting Items for Systematic Reviews and Meta-Analyses (PRISMA) 2020 guidelines for evidence synthesis, which guarantee methodological rigor, transparency, and reproducibility in the writing of reviews (52). Although the protocol was not registered in PROSPERO, all methodological decisions, including eligibility criteria, search strategy parameters, and analytic procedures, were predefined before data extraction to minimize potential bias and increase methodological transparency.

### 2.1 Eligibility criteria

The PICO(S) framework was used to clearly define the scope of the review (**Table 1**). The following criteria were used to select studies for inclusion: Non-randomized experimental studies, observational studies, and laboratory or field investigations that examined the effect of mosquito microbiota on *Plasmodium falciparum* sporozoite development, maturation, or infectivity; Studies were included if they contained *P. falciparum* data, even if they also reported on other *Plasmodium* species (e.g., *P. berghei*); Studies using only non-*P. falciparum* species were excluded; Studies involving any species of *Anopheles* mosquitoes (e.g., *An. gambiae*, *An. coluzzii*, *An. stephensi*, *An. arabiensis*, *An. funestus*, *An. moucheti*, *An. demeilloni*, *An. sinensis*), either experimentally or naturally infected with *P. falciparum*; Studies characterizing microbial communities in mosquito tissues (especially salivary glands or midgut) in relation to parasite development, sporozoite load, or transmission potential; Microbiome manipulation experiments, including: introduction of specific bacterial strains (e.g., via sugar meal feeding, microinjection); antibiotic treatment to reduce native microbiota followed by microbial re-colonization; environmental exposure experiments that affect microbiome composition; or any other intervention that alters microbial presence or abundance; Investigations reporting gene expression, immune modulation, or biochemical pathways influenced by microbial presence; Vector competence studies examining the role of microbiota in *P. falciparum* transmission dynamics; Both *in vivo* and *in vitro* approaches, and studies using either lab-reared or wild mosquito populations; Both male and female mosquitoes may be considered if relevant, though the focus is primarily on female mosquitoes, which are responsible for malaria transmission; Studies reporting one or more of the following outcomes: sporozoite load or presence in salivary glands; changes in microbiota composition; infectivity or transmission potential; immune or molecular responses; or biochemical assays relevant to sporozoite development; Studies with appropriate comparator or control groups, including: untreated/unmodified mosquitoes (native microbiota intact); antibiotic-treated mosquitoes (microbiota-depleted); mosquitoes not exposed to specific bacterial strains; different mosquito strains/species with varying microbiota; or germ-free/gnotobiotically reared mosquitoes; Both comparative and non-comparative experimental or observational studies; Only original research published in peer-reviewed journals; Studies published in English; Studies published from January 2000 to August 2026.

**Table 1:** Description of the PICO process.

PICO(S) component	Eligibility criteria
P	*Anopheles* mosquitoes infected with *P. falciparum*
I	Microbiota presence or manipulation (natural or experimental)
C	Unaltered or microbiota-depleted mosquitoes
O	Sporozoite load, prevalence, infectivity, microbiota composition, immune responses
S	Non-randomized experimental, observational, laboratory, field

Studies were included if they reported sporozoite-related outcomes in the salivary glands, either as a result of a microbiota intervention or characterization, whether conducted in the midgut or systemically. This criterion is representative of the current state of the literature, in which microbiota studies of the salivary glands are in their infancy, and the manipulation of midgut microbiota is the experimental route for studying the effects on sporozoite development. Some studies characterizing the microbiome of the human salivary glands were also included, but only a few such studies were found.

Exclusion criteria were defined to ensure methodological consistency and thematic relevance and included the following: Randomized controlled trials conducted in human or vertebrate hosts where mosquito-parasite-microbiome interactions are not assessed; Studies focusing solely on the human or vertebrate host microbiota without investigation of mosquito-associated microbial communities; Reviews, meta-analyses, editorials, opinion pieces, commentaries, conference abstracts, theses, books, or monographs without primary experimental data; Studies involving *Plasmodium* species other than *Plasmodium falciparum*; Studies involving non-*Anopheles* mosquito vectors, unless *Anopheles* is explicitly part of the experimental system; Studies that do not assess sporozoite development, load, maturation, or infectivity within the mosquito; Studies focusing exclusively on gametocyte or ookinete stages without reference to sporozoites; In silico studies or purely computational models with no biological or microbiological validation; Studies evaluating vector control or insecticide efficacy without microbiome data; Studies evaluating host immune factors without reference to microbiota; Studies focusing solely on non-bacterial exposures (e.g., insecticides, antimalarial drugs); Comparators involving non-microbiota-related interventions, such as insecticide exposure, environmental temperature shifts, genetic modifications of the mosquito unrelated to microbiome interaction, or antimalarial drug administration; Comparators across vertebrate host species (e.g., humans vs mice) that do not directly relate to mosquito microbiota effects on sporozoite development; Studies not available in English, due to limitations in translation resources and risk of misinterpretation; Articles published before January 2000; Studies where the full text is inaccessible or data are insufficient for extraction despite reasonable attempts to retrieve the article.

### 2.2 Information sources and search strategy

The following three electronic databases were used for the comprehension of the literature search: PubMed, Scopus, and Google Scholar (for grey literature and articles in early stages). The review included studies published from January 2000 to August 2026. This timeframe was selected to capture the most relevant advances in mosquito microbiome research and *Plasmodium* biology, reflecting the emergence of microbiota and vector-parasite interaction research as a recognized scientific field. The search strategy used included terms such as controlled vocabulary (e.g., MeSH terms) and free-text terms including but not limited to: “*Anopheles*”, “mosquito microbiome”, “salivary gland microbiota”, “midgut microbiota”, “*Plasmodium falciparum*”, “sporozoite development”, “vector competence”, and “paratransgenesis.” Search terms were combined by using Boolean operators (AND, OR, NOT) to narrow or expand search results. Optimization of sensitivity and precision of the search was carried out by truncation and, if available, by using database-specific filters. In order to be able to translate studies and maintain data quality, only studies published in English were included. There were no geographical restrictions. In addition, the reference list of all the included studies and relevant reviews was manually searched for other studies that might be eligible for inclusion. Rayyan was used to manage all the search results with no duplication in the results after searching.

### 2.3 Study selection

The initial search returned 1321 results (n=1321). After removing duplicates (n=228), these included 1093 publications for which titles and abstracts were screened for the inclusion criteria, which led to the selection of 55 publications for full-text review (n=55). Based on the detailed methodological and thematic eligibility criteria, 14 studies were excluded, and 41 studies (n=41) were included in the review (see PRISMA flowchart in **Figure 1**). To select studies, a two-phase selection was done, based on the guidance of the PRISMA. Screening was done independently by two reviewers. Disagreements with regard to the inclusion of studies were settled by discussion and consensus. In cases where no consensus was achieved, a third reviewer was consulted, and the decision was based on their opinion.

**Figure 1:**
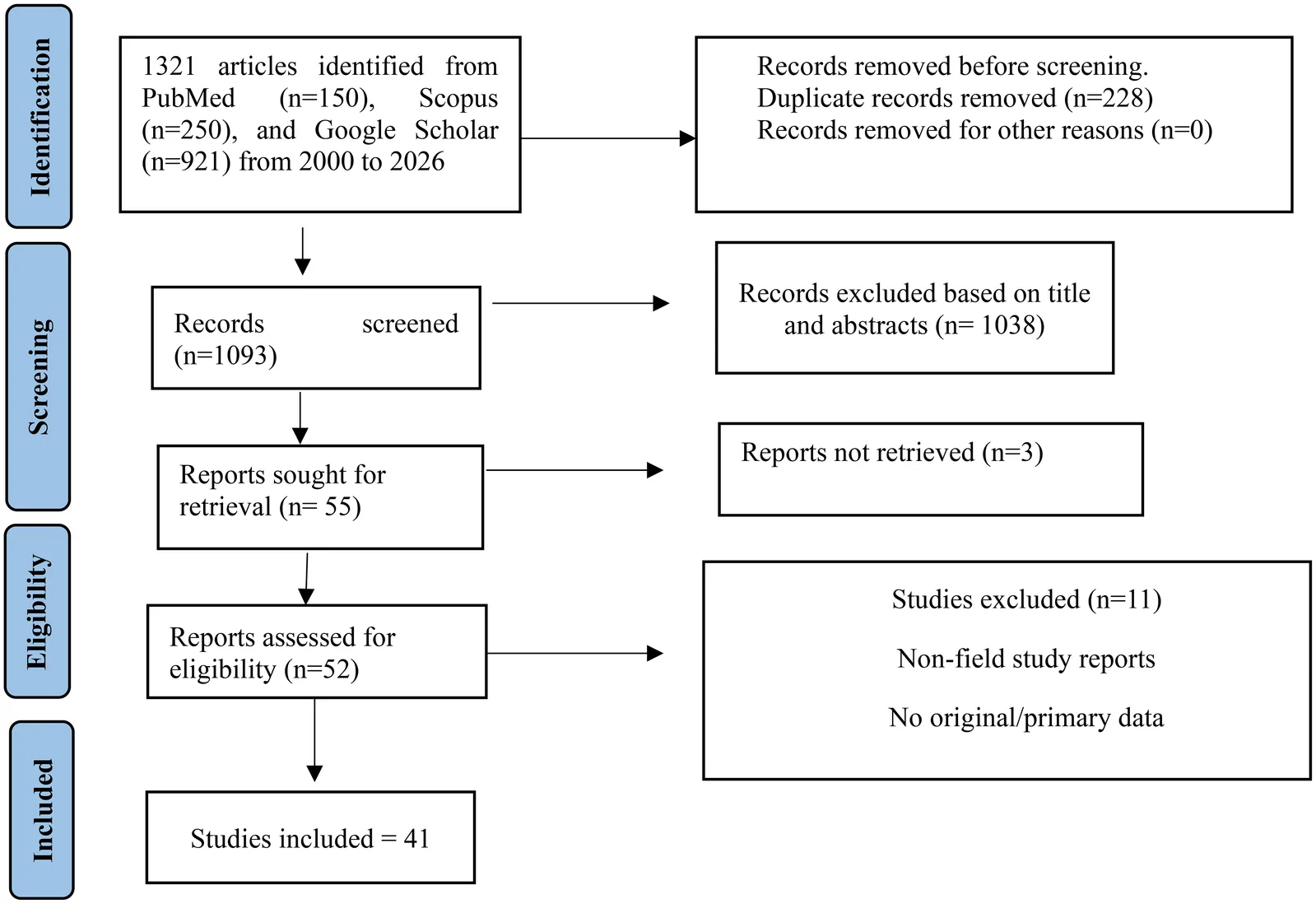
PRISMA-based systematic review process flowchart.

### 2.4 Data extraction and analysis

Study selection and data extraction were performed independently by two reviewers using a customized, predefined extraction template, with discrepancies resolved through discussion, consensus, or third-party adjudication when needed. From each study, author(s), publication year, country, study design, mosquito species and strain (noting whether laboratory-reared or wild-caught), sample characteristics (size, sex, age), microbiota characteristics (including microbial taxa identified, source, and mode of introduction), intervention type or exposure, parasite infection parameters (sporozoite load, prevalence, and infectivity), molecular or biochemical mechanisms investigated, and principal results regarding vector competence and parasite transmission were collected. For synthesis, interventions were classified according to the type of microbiota manipulation: natural microbiota characterization, *Wolbachia* infection, *Microsporidia MB* infection, *Serratia* or other bacterial supplementation, antibiotic treatment, engineered symbiotic bacteria (paratransgenesis), and immune pathway modulation studies.

Due to the significant differences in study design, mosquito species and strains, microbiota manipulation, sporozoite detection, outcome measures, and other aspects, a quantitative meta-analysis was decided *a priori* to be conducted only if there was sufficient methodological homogeneity. After study selection, the amount of clinical and methodological variation was found to be too high to allow for an aggregation of effect sizes. In view of this, a structured narrative synthesis was carried out to examine trends of effectiveness, to assess the consistency of the results from different studies and to assess whether differences in the results are related to microbial taxa, to the anatomical site of study (midgut versus salivary glands), or to the sporozoite-related outcome. Subgroup analyses were planned but were only conducted when appropriate data were available; sensitivity analyses were performed where appropriate to assess robustness of results by excluding studies with high risk of bias and/or small sample sizes.

Corresponding authors were emailed (up to two attempts over three weeks) to ask for missing, unclear, or incomplete data for studies with missing, unclear, or incomplete data. When 14 full-text articles were found that had incomplete data, the authors responded for 8, and they provided the missing data. In the other 6 studies, a response was not received, and the studies were kept as they were in the available data, with limitations stated during synthesis.

### 2.5 Risk of bias assessment

**Table 2** shows that the risk of bias was assessed across the included studies with the SYRCLE’s Risk of Bias tool (53), and that this risk was low across these studies. The overall risk of bias was generally high, but it was quite varied between domains and study designs. In all, most laboratory-based experimental studies (75%) used an appropriate sequence for the selected studies, while the majority of field-based observational studies (57%) were judged as having unclear risk for sequence generation due to the non-applicability of randomization. The majority of studies (80%) showed comparable baseline characteristics, particularly in laboratory experiments, while field studies were rated as unclear risk due to inherent variability in wild-caught populations. Allocation concealment was reported in only 40% of studies, and random housing was documented in approximately 65%. Blinding of caregivers and outcome assessors was a major limitation, with only 20% of studies reporting adequate blinding, resulting in a high risk of performance and detection bias in most studies. Attrition bias was low in 85% of studies due to clear reporting of mosquito mortality and exclusion of unfed mosquitoes, and selective outcome reporting was low in 90% of studies. Other potential sources of bias included small sample sizes (n < 20 per group) in 35% of studies, lack of power calculations (60%), reliance on single experimental replicates (25%), and potential off-target effects of antibiotic treatments on mosquito physiology or parasite development.

**Table 2:** Summary of risk of bias assessment (SYRCLE’s RoB tool).

Domain	Low risk (%)	Unclear risk (%)	High risk (%)
Sequence generation	55	40	5
Baseline characteristics	65	30	5
Allocation concealment	25	50	25
Random housing	50	35	15
Blinding of caregivers	15	40	45
Blinding of outcome assessors	20	35	45
Incomplete outcome data	75	20	5
Selective outcome reporting	80	15	5
Other sources of bias	50	30	20

For field-based observational studies, domains requiring randomization (sequence generation, allocation concealment) were rated as ‘unclear risk’ as randomization did not apply to these study designs. The high risk of bias in blinding domains is a recognized limitation in vector biology research, where technical constraints often prevent complete blinding.

### 2.6 Assessment of heterogeneity and feasibility of meta-analysis

Following data extraction, the degree of heterogeneity across studies was assessed to determine whether a quantitative meta-analysis was feasible. Studies varied considerably across multiple domains: Mosquito species: Eight different *Anopheles* species were studied (*An. gambiae*, *An. coluzzii*, *An. stephensi*, *An. arabiensis*, *An. funestus*, *An. moucheti*, *An. demeilloni*, *An. sinensis*), with substantial biological differences in vector competence and microbiota composition; Study designs: Studies included laboratory-based experimental designs (n = 22), field-based observational studies (n = 11), and combined approaches (n = 8), each with different levels of experimental control and inherent biases; Microbiota manipulation methods: Approaches included antibiotic treatment (n = 10), bacterial supplementation via sugar or blood meals (n = 12), genetic modification of bacteria (paratransgenesis, n = 3), RNAi-mediated gene silencing (n = 8), and CRISPR-Cas9 gene knockout (n = 2); Outcome measures: Studies reported a range of outcomes including sporozoite load (measured by microscopy, qPCR, ELISA, or immunofluorescence), sporozoite prevalence, oocyst intensity, ookinete formation, immune gene expression, and microbiota composition. Measurement units and timing varied substantially; Microbiota types: Studies investigated different microbial taxa including *Wolbachia* (n = 9), *Microsporidia* MB (n = 8), *Serratia* spp. (n = 5), *Enterobacter* spp. (n = 2), *Asaia* spp. (n = 1), *Pantoea* spp. (n = 1), *Chromobacterium* (n = 1), *E. coli* (n = 1), and whole microbial communities (n = 3). Due to this substantial methodological and clinical heterogeneity, a quantitative meta-analysis was not feasible. A narrative synthesis was therefore conducted as prespecified in the protocol.

## 3 Results

### 3.1 Study characteristics

A summary of the 41 included studies is presented in **Table 3**. These studies were published between 2009 and 2026, with most of them published after 2015, indicating a recent growing interest in the study of mosquito microbiota over the past decade. The studies were conducted in 15 different countries across Africa, Asia, Europe, and North America, with Burkina Faso having the highest representation (n = 9), followed by Cameroon (n = 6), Kenya (n = 5), and Benin (n = 4).

**Table 3:** Summary characteristics of included studies.

Author (year)	Country	Mosquito species	Study design	Microbiota focus	Key outcome(s)
Meister et al., 2009 (54)	UK/Cameroon/France/Greece/Germany	*An. gambiae*	Laboratory experimental	PGRPLC, bacteria	PGRPLC silencing increased *P. berghei* oocyst intensity 4.4-fold (P<0.001) and significantly increased *P. falciparum* oocyst intensity (P<0.005) and prevalence (from 41% to 52%).
Dong et al., 2009 (55)	USA	*An. gambiae*	Laboratory experimental	Midgut microbiota, antibiotics	Antibiotic treatment increased *P. falciparum* susceptibility.
Cirimotich et al., 2011 (21)	Zambia	*An. gambiae*, *An. stephensi*, *An. arabiensis*	Laboratory experimental	*Enterobacter* Esp_Z	Esp_Z reduced *P. falciparum* ookinete formation by 98% and oocyst development by 99% via reactive oxygen species (ROS) production.
Hughes et al. (2011) (56)	USA	*An. gambiae*	Laboratory experimental	*Wolbachia* wMelPop, wAlbB	Somatic *Wolbachia* inhibited *P. falciparum*; wMelPop virulent post-bloodmeal
Wang et al., 2012 (35)	USA	*An. gambiae*, *An. stephensi*	Laboratory experimental	Engineered *Pantoea* (paratransgenesis)	Scorpine and EPIP reduced *P. falciparum* oocysts by ~98%, prevalence by 84%
Boissiere et al., 2012 (18)	Cameroon	*An. gambiae*	Field observational	Midgut microbiota (16S pyrosequencing)	*Enterobacteriaceae* abundance correlated positively with *P. falciparum* infection.
Blumberg et al., 2013 (57)	USA	*An. gambiae*	Laboratory experimental	Bacteria-independent immune defenses	SRPN7 and CLIPC2 identified; SRPN7 silencing reduced *P. falciparum* infection, indicating SRPN7 is a negative regulator of anti-*Plasmodium* immunity.
Bian et al., 2013 (45)	USA	*An. stephensi*	Laboratory experimental	*Wolbachia* wAlbB (stable transinfection)	Stable wAlbB reduced *P. falciparum* oocysts and sporozoites; CI demonstrated.
Ramirez et al., 2014 (58)	USA	*An. gambiae*, *Ae. aegypti*	Laboratory experimental	*Chromobacterium* Csp_P	Csp_P reduced *P. falciparum* infection; entomopathogenic
Bahia et al., 2014 (23)	USA	*An. gambiae*	Laboratory experimental	*Serratia marcescens* and others	S. *marcescens* most effective; *in vitro*/*in vivo* inhibition; secreted factors
Gendrin et al., 2015 (59)	UK/Burkina Faso	*An. gambiae*	Laboratory experimental	Antibiotics (penicillin/streptomycin)	Antibiotics increased *P. falciparum* infection and mosquito survival/fecundity.
Gendrin et al., 2016 (60)	UK/Burkina Faso	*An. coluzzii*, *An. gambiae*	Laboratory experimental	Antibiotics (azithromycin, doxycycline, co-trimoxazole)	Azithromycin reduced *P. falciparum* infection; doxycycline increased infection at high concentrations
Shaw et al., 2016 (61)	Burkina Faso	*An. coluzzii*	Field observational	*Wolbachia* wAnga-BF	Negative correlation between *Wolbachia* and *Plasmodium* development; 0.8% *Plasmodium* in W+ vs 10.4% in W−; *Wolbachia* accelerates egg laying but does not induce cytoplasmic incompatibility.
Tchioffo et al., 2016 (62)	Cameroon/France	*An. coluzzii*	Laboratory + Field	*E. coli* ST95 vs ST73	*E. coli* ST95 (444) inhibited *P. falciparum*; ST73 had no effect; strain-specific.
Gendrin et al., 2017 (63)	UK/Cameroon	*An. coluzzii*	Laboratory experimental	PGRPLA, PGRPLB	PGRPLA positively regulates the Imd pathway; PGRPLB negatively regulates it.
Gomes et al., 2017 (64)	Mali	*An. gambiae*, *An. coluzzii*	Laboratory + Field	*Wolbachia* wAnga-Mali	wAnga-Mali negative correlation with *P. falciparum* sporozoites; stage-specific effect.
Song et al., 2018 (65)	China	*An. stephensi*	Laboratory experimental	PGRP-LD	PGRP-LD regulates microbiota homeostasis and peritrophic matrix integrity; knockdown increases *P. berghei* infection.
Bassene et al., 2018 (66)	Senegal	*An. gambiae*, *An. funestus*	Field observational	Midgut microbiota (16S metagenomics)	*Burkholderia* abundant in uninfected mosquitoes; microbiota more diverse in infected samples.
Herren et al., 2020 (36)	Kenya	*An. arabiensis, An. gambiae*	Laboratory + Field	*Microsporidia* MB	*Microsporidia* MB blocks *P. falciparum* sporozoites; vertical transmission; no fitness cost.
Inbar et al., 2021 (67)	USA	*An. stephensi*	Laboratory experimental	LRIM1 knockout (CRISPR-Cas9)	LRIM1 KO increased bacterial load; reduced *P. falciparum* infection in non-aseptic conditions; fecundity compromised.
Gao et al., 2021 (33)	China	*An. sinensis*, *An. stephensi*, *An. gambiae*	Laboratory experimental + Field	*Serratia ureilytica* Su_YN1 (AmLip)	AmLip lipase kills *Plasmodium*; vertical/horizontal transmission; spreads through populations.
Huang et al., 2022 (68)	USA	*An. stephensi*	Laboratory experimental	Transgenesis + paratransgenesis (*Serratia* AS1-multi)	Transgenic + paratransgenic combination gave 93% transmission blocking in a mouse model.
Habtewold et al., 2022 (69)	UK	*An. coluzzii*	Laboratory experimental	Circadian clock, microbiota	Circadian rhythm modulates *P. falciparum* infection; higher infectivity at night.
Akorli et al., 2022 (70)	Ghana/UK	*An. gambiae*	Laboratory experimental	*Serratia marcescens* bio-products	S. *marcescens* spent media reduced *P. falciparum* prevalence and oocyst intensity.
Mouillaud et al., 2023 (47)	Cameroon	*An. moucheti*	Field observational	*Wolbachia* wAnmo	Near-fixation *Wolbachia* (95%) but NO correlation with *P. falciparum.*
Gao et al., 2023 (71)	China	*An. stephensi*, *An. gambiae*	Laboratory experimental	*Serratia* OMVs (AmLip delivery)	AmLip-loaded OMVs target *Plasmodium* via the phosphatidylcholine scavenging pathway.
Llergo et al., 2024 (72)	Spain	*An. stephensi*	Laboratory experimental	Artificial light at night	Photophase significantly reduced oocyst intensity; microbiota enhanced the effect.
Onchuru et al., 2024 (73)	Kenya	*An. arabiensis*	Laboratory experimental	*Microsporidia* MB vertical transmission	Germline stem cell infection; 97% transmission to follicles; no fitness cost.
Vandana et al., 2024 (46)	USA	*An. stephensi*	Laboratory experimental	*Wolbachia*-regulated immune genes	TEP4, TEP15, lysozyme C2, CLIPB2, CLIPB4, PGRP-LD suppress *P. falciparum.*
Ahouandjinou et al., 2024 (74)	Benin	*An. gambiae*, *An. coluzzii*	Field observational	*Wolbachia*, *Microsporidia* MB	First report in Benin; *Microsporidia* prevalence 41-57%; *Wolbachia* 1.3-5.1%.
Millogo et al., 2024 (75)	Burkina Faso	*An. coluzzii*, *An. gambiae, An. arabiensis*	Laboratory + Field	*Microsporidia* MB transmission	Transgenerational and sexual transmission; negative correlation with *P. falciparum.*
Moustapha et al., 2024 (76)	Niger	*An. coluzzii*, *An. gambiae*, *An. arabiensis*	Field observational	*Microsporidia* MB	First identification in Niger; prevalence 6.8%; only in *An. coluzzii.*
Tatsinkou Maffo et al., 2024 (77)	Cameroon	*An. gambiae*, *An. coluzzii, An. arabiensis*	Field observational	*Asaia*	No correlation between *Asaia* and *P. falciparum*; species-specific patterns.
Estelle et al., 2025 (78)	Burkina Faso	*An. coluzzii*, *An. gambiae, An. arabiensis*	Laboratory + Field	*Wolbachia* w-Anga	Unstable vertical transmission (10-16%); negative correlation with *P. falciparum.*
Sandeu et al., 2025 (79)	Cameroon	*An. gambiae*	Field observational	Microbiota diversity (16S rRNA)	14 genera more abundant in uninfected mosquitoes; 2 in infected; seasonal variation.
Aliyu et al., 2025 (80)	Nigeria	*An. gambiae*	Field observational	*Microsporidia*	First report in Nigeria; 12% prevalence; no co-infection with *P. falciparum* or *W. bancrofti.*
Traor� et al., 2026 (34)	Burkina Faso	*An. coluzzii*, *An. gambiae*, *An. arabiensis*	Field observational	*Serratia* spp.	Prevalence 13.3%; present in ovaries, midgut, spermatheca, testes; phylogenetically distinct.
Sandeu et al., 2026 (81)	Cameroon	*An. coluzzii*, *An. gambiae*, *An. arabiensis*	Field observational	*Microsporidia* MB	Prevalence 1.13%; vertical transmission 50-100%; no *P. falciparum* co-infection.
Sougoufara et al., 2026 (82)	DRC	*An.* *demeilloni*	Field observational	*Wolbachia* wAnD	wAnD correlated with lower sporozoite rates (significant for abdominal infection) and with altered microbiome composition, but not with reduced microbiome diversity.
Mmweteni et al., 2026 (48)	Tanzania	*An. funestus*	Field observational	*Wolbachia* supergroup B	First report in *An. funestus*; prevalence 46.5-70.8%; low *P. falciparum* co-infection
Sovi et al., 2026 (83)	Benin	*An. gambiae, An. coluzzii*	Field observational	*Microsporidia MB*	No *P. falciparum* infection in any *Microsporidia MB*-positive mosquito

### 3.2 Mosquito species

Various species of *Anopheles* were studied in the included studies. The most frequently studied species were *An. gambiae* (n = 18), *An. coluzzii* (n = 15), *An. stephensi* (n = 9), *An. arabiensis* (n = 7), *An. funestus* (n = 4), *An. moucheti* (n = 2), *An. demeilloni* (n = 1), and *An. sinensis* (n = 1). Multiple species were studied in several studies, and seven studies compared two or more *Anopheles* species.

### 3.3 Study design

The studies included in the analysis ranged from laboratory-based experimental studies (n=22) to field-based observational studies (n=11) and combined laboratory–field approaches (n=8). Of the experimental studies, microbiota manipulation was performed by various methods, such as antibiotic treatment (n=10), bacterial supplementation using sugar or blood meals (n = 12), genetic modification of bacteria (paratransgenesis, n=3), RNAi-mediated gene silencing (n=8), and CRISPR-Cas9 gene knockout (n=2).

### 3.4 Microbiota types investigated

A wide range of microbial taxa from *Anopheles* mosquitoes were studied. These included bacteria like *Wolbachia* (n=12), *Serratia* spp. (n=6), *Enterobacter* spp. (n=4), *Asaia* spp. (n=4), *Pantoea* spp. (n=2), *Chromobacterium* (n=1), *Escherichia coli* (n = 1); microsporidian fungi (*Microsporidia MB*, n = 6). Whole microbial communities were studied in several metagenomic studies (n = 6).

### 3.5 Synthesis of findings

The included studies were synthesized narratively according to key themes: (1) *Wolbachia* infection and *Plasmodium* interactions; (2) *Microsporidia* MB and transmission blocking; (3) *Serratia* and other bacterial species with anti-*Plasmodium* activity; (4) Engineered symbionts and paratransgenesis; (5) Immune pathways and host responses; (6) Microbiota composition and vector competence; (7) Environmental factors; (8) Antibiotics and microbiota disruption; and (9) Combined strategies for transmission blocking.

#### 3.5.1 *Wolbachia* infection and *Plasmodium* interactions

*Wolbachia* appeared to be the most extensively studied microbial symbiont, with 12 included studies investigating its association with *Plasmodium* infection in various *Anopheles* species. The results showed that the impact of *Wolbachia* on the development of *P. falciparum* is strain- and species-specific, as shown in **Table 4**.

**Table 4:** Summary of *Wolbachia* strains and their effects on *Plasmodium* infection.

Strain	Host species	Setting	*P. falciparum* effect	Key finding
wAlbB	*An. stephensi*	Stable transinfection	↓ Oocysts, ↓ Sporozoites	CI, ROS-mediated; 3.4-3.7x sporozoite reduction
wMelPop/wAlbB	*An. gambiae*	Somatic infection	↓ Oocysts (40-60%)	Virulent post-bloodmeal; immune dynamic
wAnga-Mali	*An. gambiae*, *An. coluzzii*	Natural (Mali)	↓ Sporozoites, ↑ Oocysts	Stage-specific; 46-95% prevalence; no CI
wAnga-BF	*An. coluzzii*	Natural (Burkina Faso)	Negative correlation	0.86% *Plasmodium* in W+ vs 10.48% in W-
w-Anga	*An. coluzzii*, *An. gambiae*	Natural (Burkina Faso)	Negative correlation	4.59% in W+ vs 22.02% in W-; unstable transmission
wAnD	*An. demeilloni*	Natural (DRC)	Negative correlation	Only in W- cephalothoraxes; different microbiome composition
wAnmo	*An. moucheti*	Natural (Cameroon)	NO correlation	95% prevalence; still a major vector
wAnga	*An. gambiae*, *An. coluzzii*	Natural (Benin)	No co-infection observed	Low prevalence (1.3-5.1%); first report from Benin
Supergroup B	*An. funestus*	Natural (Tanzania)	Not significant	46.5-70.8% prevalence; low co-infection

↓ means decrease, while ↑ means increase.

##### 3.5.1.1 Stable transinfection studies

With the success of their transinfection of the wAlbB strain into *An. stephensi*, Bian et al. (45) established the first stable *Wolbachia* infection in an *Anopheles* species, which showed perfect maternal transmission and high cytoplasmic incompatibility (CI). *P. falciparum* oocyst prevalence and intensity were significantly reduced in the presence of wAlbB infection, and even greater inhibition was observed at the sporozoite stage (3.4- and 3.7-fold reduction in sporozoite loads). The inhibition was associated with higher levels of reactive oxygen species (ROS) in wAlbB-infected mosquitoes, which suggests a mechanistic role for oxidative stress in parasite suppression.

##### 3.5.1.2 Somatic infections

Somatic infections of two strains of *Wolbachia* (wMelPop and wAlbB) in *An. gambiae* were characterized by Hughes et al. (56). *P. falciparum* oocyst development was significantly inhibited by both strains (40-60%). The mosquito immune response was dynamic: initial immune induction was followed by suppression coincident with *Wolbachia* replication. The wMelPop strain was especially virulent, resulting in high mortality 12–24 hours post-bloodmeal, and 80% mortality by day 3. This acute mortality implied potential in field settings for *Wolbachia*-induced post-bloodmeal mortality to impact transmission potential.

##### 3.5.1.3 Naturally occurring infections

###### 3.5.1.3.1 Burkina Faso (wAnga-BF)

Shaw et al. (61) found natural *Wolbachia* infections (wAnga) and a negative correlation of the presence of *Wolbachia* with *Plasmodium* development in samples of *An. coluzzii* from Burkina Faso. Only 0.8% of *Wolbachia*-infected-fed mosquitoes were found to be carrying *Plasmodium* compared to 10.4% of mosquitoes in a study of 221 blood-fed mosquitoes. The wAnga-BF strain, however, did not induce cytoplasmic incompatibility under laboratory conditions.

###### 3.5.1.3.2 Mali (wAnga-Mali)

Gomes et al. (64) found a different *Wolbachia* strain (wAnga-Mali) in populations of *An. gambiae* and *An. coluzzii* from Mali. The results of the phylogenetic analysis showed that wAnga-Mali clusters with supergroup A *Wolbachia* and shares only 99.8% identity with the *Wolbachia* of cat fleas (*Ctenocephalides*), suggesting a horizontal transfer. Field surveys showed that sporozoite prevalence of *P. falciparum* was significantly lower in *Wolbachia*-infected females (29%) compared to uninfected females (65%, P < 0.0001), with lower intensity of sporozoite infection. Laboratory infections with an *An. coluzzii colony* derived from Malian mosquitoes showed a surprising stage-specific effect: wAnga-Mali had a slightly enhanced oocyst infection but significantly reduced the prevalence of sporozoite infection (18% in W+ vs 44% in W−, P < 0.004) and the intensity (P < 0.01). This suggests that *Wolbachia* has a negative effect on sporozoite development, rather than early midgut stages.

###### 3.5.1.3.3 Cameroon/DRC (wAnD)

Sougoufara et al. (82) analyzed the wAnD *Wolbachia* strain from wild *An. demeilloni* collected in the Democratic Republic of Congo. *Wolbachia* prevalence was significantly higher in abdomens (80%) than cephalothoraxes (17%), which is consistent with maternal transmission. The overall prevalence of sporozoites was quite low (1.3%), and all mosquitoes carrying *P. falciparum* were detected in *Wolbachia*-negative cephalothoraxes. 16S rRNA amplicon sequencing revealed significant differences in microbiome composition between *Wolbachia*-positive and -negative cephalothoraxes (P < 0.05), but no significant difference in overall microbiome diversity (alpha diversity) was observed. A negative correlation was found between wAnD presence in abdomens and sporozoite rates (P = 0.0329), suggesting a possible transmission-blocking effect.

###### 3.5.1.3.4 Benin (wAnga)

Ahouandjinou et al. (74) found the first natural *Wolbachia* infection in *An. gambiae* in Benin. The prevalence of *Wolbachia* in *An. gambiae* and *An. coluzzii* was 5.1% and 1.3%, respectively, with a mean of 2.5% across the species complex. The rate of sporozoites was 0.74%, and there was no co-infection of *P. falciparum* with *Wolbachia* in mosquitoes.

###### 3.5.1.3.5 Tanzania (*An. funestus*)

The first report of *Wolbachia* in wild *An. funestus* s.l. in Tanzania was recently reported by Mmweteni et al. (48). *Wolbachia* prevalence was high, with 46.5% and 70.8% of samples found positive, respectively, by primary and nested PCR, compared to the prevalence found previously in *An. arabiensis* in the same region. Phylogenetic analysis of 16S rDNA, COXA, and wsp genes confirmed that the detected strains clustered with supergroup B *Wolbachia*. Only three mosquitoes carried *P. falciparum* sporozoites (prevalence 0.8%), with only one showing co-infection (0.3%). There was a potential negative association, as the observed co-infection prevalence (0.3%) was lower than expected (0.4–0.5%), but the low P. falciparum prevalence prevented a statistically significant conclusion.

###### 3.5.1.3.6 Burkina Faso (w-Anga)

In Burkina Faso, the prevalence of the w-Anga strain in wild *Anopheles* was 13.84% (higher in *An. coluzzii* (21.78%) than in *An. gambiae* s.s. (16.89%)), and the *Plasmodium*-blocking potential of the w-Anga strain was investigated by Estelle et al. (78). The results of the phylogenetic analysis indicated that w-Anga belonged to supergroup B. The prevalence of *P. falciparum* infection was significantly lower in w-Anga-positive females compared to w-Anga-negative females (4.59% vs 22.02%, respectively, P = 0.00033). However, the vertical transmission rate was unstable, with a 10.64% transmission rate from F0 to F1 and 16.67% from F1 to F2, limiting its potential for sustainable control applications. In contrast, a fitness advantage appears to be the case for w-Anga-infected females, as they produced significantly more eggs than uninfected females (56.63 vs 42.42; P = 0.019).

###### 3.5.1.3.7 Cameroon (*An. moucheti*)

Mouillaud et al. (47) studied *Wolbachia* in *An. moucheti*, which is the major malaria vector of Central Africa. *Wolbachia* prevalence was close to fixation (95%), and there was no correlation between *Wolbachia* infection and *P. falciparum* sporozoite prevalence. In fact, *Wolbachia* density was slightly higher in *P. falciparum*-infected mosquitoes (P = 0.004), and there was no significant correlation between *Wolbachia* and *P. falciparum* densities (R = -0.11, P = 0.49). This suggests that in long-established *Wolbachia*-host associations, the protective phenotype may be absent, which is consistent with the role of *An. moucheti* as a major malaria vector in Central Africa despite high *Wolbachia* prevalence.

##### 3.5.1.4 *Wolbachia*-induced immune genes

Vandana et al. (46) used transcriptomic analysis of wAlbB-infected *An. stephensi* to find immune genes that are responsive to *Wolbachia* infection. RNA-seq of midguts and carcasses from sugar-fed and blood-fed mosquitoes identified 10 upregulated and 14 downregulated putative immune genes in midguts, and 31 upregulated and 15 downregulated in carcasses at 24 hours post-blood feeding. Functional studies using RNAi silencing demonstrated that eight *Wolbachia*-regulated genes significantly increased *P. falciparum* infection intensity when silenced: TEP4, TEP15, lysozyme C2, CLIPB2, CLIPB4, PGRP-LD, and two novel genes (peritrophin-44-like and macrodomain-encoding). Based on these results, *Wolbachia* infection can be used to modulate mosquito immunity, reducing *Anopheles* permissiveness for *Plasmodium* infection.

#### 3.5.2 *Microsporidia MB* and transmission blocking

The microsporidian symbiont, *Microsporidia MB*, a vertically transmitted microsporidian symbiont, was identified in six included studies across multiple African countries, demonstrating consistent transmission-blocking potential as shown in **Table 5**.

##### 3.5.2.1 Discovery and characterization

*Microsporidia MB* was first reported from geographically isolated populations of *An. arabiensis* in Kenya, with 0- 9% prevalence by Herren et al. (36). Phylogenetic analysis placed *Microsporidia MB* in clade IV of the *Microsporidia*, with 97% similarity to *Crispospora chironomi*. The infection rates varied seasonally, with the highest rates of infection being observed 4–6 weeks after peak rainfall. *Microsporidia MB* was easily transmitted vertically (45-100%) and exhibited no significant fitness-impairing effects (no reduction in fecundity or adult survival). Importantly, field-collected *Microsporidia MB*-infected *An. arabiensis* were also negative for *P. falciparum* gametocytes, while experimental infection results indicated a lack of detection of sporozoites in *Microsporidia MB*-infected mosquitoes. Immune and digestive genes were found to be upregulated during RNA sequencing, such as cecropin-2, gambicin, and antryp serine proteases, which implies immune priming as a mechanism. The absence of *Plasmodium* in both midguts (oocysts) and head-thorax (sporozoites) indicated that *Microsporidia MB* impairs *Plasmodium* development before oocyst formation.

##### 3.5.2.2 Vertical transmission mechanism

Onchuru et al. (73) explained the mechanistic basis of *Microsporidia MB* vertical transmission in *An. arabiensis*. *Microsporidia MB* was found in the germarium by fluorescence *in situ* hybridization (FISH) microscopy and infects the germline stem cells (GSCs), which are then passed to cystocytes during stem cell division. In the nurse cells, *Microsporidia MB* infection intensity increases, followed by an increase in the oocytes via active symbiont cell division of the symbionts present within the cells. The efficiency of transmission from stem cell to follicle was 97%, while adult-to-adult vertical transmission was only 61-63%, suggesting that some of the offspring lose their symbiont during development. In the case of *Microsporidia MB*, proliferation was observed in the oocyte after blood feeding, indicating coordination with egg development. This is a highly specialized vertical transmission strategy which accounts for host specificity and the absence of fitness costs.

##### 3.5.2.3 Field Prevalence in West Africa

###### 3.5.2.3.1 Burkina Faso

Millogo et al. (75) analyzed the prevalence and transmission of *Microsporidia MB* in mosquitoes of the *An. gambiae* complex from Burkina Faso. Prevalence was significantly higher in *An. coluzzii* (21.78%) than *An. gambiae* s.s. (16.89%, P = 0.03). Sexual transmission was demonstrated: infected males transmitted *Microsporidia MB* to uninfected females (9.23% infection rate), and infected females transmitted to uninfected males (33.33%). Transgenerational transmission was observed across F0-F3 generations, with a mean prevalence of 22.19% in *An. coluzzii* and 30.23% in *An. gambiae* s.s. (P < 0.001). A significant negative correlation was observed between *Microsporidia MB* and *P. falciparum* infection, with 73% of *Microsporidia MB*-positive mosquitoes negative for *Plasmodium* (P < 0.01).

###### 3.5.2.3.2 Cameroon

In Cameroon, the first field evidence of *Microsporidia MB* was provided by Sandeu et al. (81). The prevalence was 1.13% overall, with only *An. coluzzii* from Gounougou. Longitudinal analysis showed a declining prevalence from 2.72% (2019) to 0.98% (2024). Phylogenetic analysis showed that the sequence was closely related to Kenyan strains with 99.22% similarity. Vertical transmission from F0 to F1 ranged from 50-100%. In particular, none of the *Microsporidia MB*-positive mosquitoes were *P. falciparum* positive.

###### 3.5.2.3.3 Niger

In Niger, the presence of *Microsporidia MB* has been observed in *An. coluzzii* for the first time from Zinder City by Moustapha et al. (76). Prevalence: 6.8% (17/251), exclusively in *An. coluzzii*. Only one mosquito was PCR-positive for *P. falciparum*, and this mosquito was *Microsporidia MB*-negative. This extends the geographical range of *Microsporidia MB* to the Sahelian zone of West Africa.

###### 3.5.2.3.4 Benin

Ahouandjinou et al. (74) reported high *Microsporidia MB* prevalence in Benin: 41.0% in *An. gambiae* s.s. and 57.0% in *An. coluzzii*, with a mean of 53.4% across the species complex. This is the highest prevalence reported to date, suggesting potential regional variation. However, the sporozoite rate was low (0.74%), and only one *Microsporidia MB*-positive mosquito was found to carry *P. falciparum*. The study also reported that *An. coluzzii* was the predominant species (93.7%), and the L1014F *kdr* mutation frequency was 75.8% across the species complex.

###### 3.5.2.3.5 Nigeria

In Nigeria, Aliyu et al. (80) reported the prevalence of Microsporidia in *An. gambiae* s.l. at 12% from Ahmadu Bello University, Zaria, Nigeria. Co-infections of *Microsporidia* with *P. falciparum* or *W. bancrofti* were not seen. This study was able to establish *Microsporidia MB* in Nigeria for the first time and showed its possible role as an antagonist in the development of pathogens.

##### 3.5.2.4 *kdr* mutation association

Sovi et al. (83) conducted a study on *An. gambiae* s.l. from Benin to test the association between *Microsporidia MB* infection and the L1014F *kdr* mutation. The prevalence of *Microsporidia MB* in *An. coluzzii* and *An. gambiae* s.s. was 1.6% and 2.2%, respectively. *Microsporidia MB* was not found in any *kdr*-SS, but was found in both *kdr*-RS (1.9%) and *kdr*-RR (1.9%) genotypes. The mean load of *Microsporidia MB* DNA was significantly higher in *kdr*-RR (23.23 ng/µl) than in *kdr*-RS (10.13 ng/µl). Ecological niche modelling identified elevation (78%) and soil (20%) as the main environmental drivers of *Microsporidia MB* habitat suitability. All the *Microsporidia MB* positive mosquitoes were negative for *P. falciparum* sporozoites.

**Table 5:** *Microsporidia MB* prevalence and transmission-blocking evidence across African countries.

Country	Host Species	Prevalence	*P. falciparum* co-infection	Key finding
Kenya	*An. arabiensis*	0-9%	None	Blocks sporozoites; no fitness cost; vertical transmission 45-100%
Burkina Faso	*An. coluzzii*, *An. gambiae*	16-21%	Negative correlation	Sexual transmission; transgenerational; 73% M+ mosquitoes Pf-negative
Cameroon	*An. coluzzii*	1.13%	None	Vertical transmission 50-100%; closely related to Kenyan strains
Niger	*An. coluzzii*	6.8%	None	First reported in Sahelian West Africa
Benin	*An. gambiae*, *An. coluzzii*	41-57%	None	Highest reported prevalence
Nigeria	*An. gambiae*	12%	None	First report from Nigeria; no co-infection with *W. bancrofti*

#### 3.5.3 *Serratia* and other bacterial species with anti-*Plasmodium* activity

##### 3.5.3.1 *Serratia ureilytica* Su_YN1 and AmLip

Gao et al. (33) identified *Serratia ureilytica* Su_YN1 from wild *An. sinensis* in Yunnan province, China, where local malaria cases were significantly lower than imported cases despite high vector abundance. Su_YN1 colonized mosquito midguts and made mosquitoes resistant to *P. falciparum* and *P. berghei* infection. The bacterium can produce a 65 kDa lipase (AmLip) which is directly lethal against parasites at various stages. AmLip knockout abolished antimalarial activity, while complementation restored it. Su_YN1 is transmitted vertically (via the egg surface), transstadially (larvae to adults), and sexually (male to female). Introduction of 5% of males infected with Su_YN1 resulted in a 100% infection rate of progeny in cage experiments, and the infection has been transmitted for three successive generations. This natural symbiont may be used as a potential malaria control without using GMOs.

##### 3.5.3.2 Outer membrane vesicle-mediated delivery

The mechanism of AmLip delivery to *Plasmodium* parasites was also explained by Gao et al. (71). Host serum triggers a high release of outer membrane vesicles (OMVs) and the antimalarial effector protein AmLip in the mosquito gut by stimulating Su_YN1. AmLip is first secreted via the type I secretion system (TISS) and then preferentially loaded onto OMVs. Of particular interest, the serum-induced OMVs contain some of the serum-derived lipids, including phosphatidylcholine (PC), which is essential for the uptake of OMVs by Plasmodium through the PC scavenging pathway. This selective targeting causes the parasites to be killed without affecting healthy blood cells. OMVs from AmLip-knockout strains lacked antimalarial activity, confirming that AmLip binding confers the anti-*Plasmodium* effect.

##### 3.5.3.3 *Serratia marcescens* bio-products

This study by Akorli et al. (70) focused on bio-products of Ghanaian *An. gambiae*-isolated *Serratia marcescens* and *Enterobacter cloacae*. Spent media from S. *marcescens* cultures significantly reduced both *P. falciparum* infection prevalence (P = 4.02×10^-5^) and oocyst intensity (P<2×10^-16^) when added to infectious blood meals. *E. cloacae* products showed no significant effect, indicating strain-specific activity. This implies that secreted factors of *S. marcescens* inhibit parasites in a manner that does not involve mosquito immune activation.

##### 3.5.3.4 *Serratia* in Burkina Faso

Traoré et al. (34) molecularly identified *Serratia* spp. in *An. gambiae* complex mosquitoes collected from the wild in Burkina Faso. Overall prevalence was 13.3%, with higher prevalence in adult mosquitoes (11.4%) than larvae (1.9%, P = 0.03), and in females (12.4%) than males (2.3%, P = 0.01). *Serratia* was found in the ovaries (60%), midgut (55%), spermatheca (55%), and salivary glands (10%), indicating several routes of transmission. Phylogenetic analysis found that the strains from Burkina Faso clustered separately from strains reported in neighboring countries (Ghana, Nigeria). There was only one mosquito infected with both *Serratia* and *P. falciparum*, with a possible negative association.

**Table 6** summarizes the key anti-plasmodial bacterial species identified in the reviewed literature.

**Table 6:** Key anti-*Plasmodium* bacterial species and mechanisms.

Bacterial species	Source	Anti-*Plasmodium* mechanisms	Transmission	Key finding
*Serratia ureilytica* Su_YN1	China (*An. sinensis*)	AmLip lipase via OMVs; PC scavenging pathway	Vertical, sexual, transstadial	98% inhibition; spreads through populations
*Serratia marcescens*	Ghana (*An. gambiae*)	Secreted bio-products	Not specified	Reduced prevalence and oocyst intensity
*Serratia* spp.	Burkina Faso	Not determined	Present in multiple organs	13.3% prevalence; low co-infection with *P. falciparum*
*Enterobacter* Esp_Z	Zambia (*An. arabiensis*)	ROS production	Not specified	99% inhibition; IMD-independent
*Chromobacterium* Csp_P	Panama (*Ae. aegypti*)	Biofilm-associated factors	Not specified	Reduces *P. falciparum* and dengue; entomopathogenic
*Pantoea agglomerans* (engineered)	USA	Scorpine, EPIP secretion	Not specified	98% oocyst reduction; 84% prevalence reduction

#### 3.5.4 Immune pathways and host responses

##### 3.5.4.1 PGRPLC and IMD pathway

By demonstrating that PGRPLC is a key mediator of antibacterial defense in An. gambiae through peptidoglycan recognition of both Gram-positive and Gram-negative bacteria, Meister et al. (54) have provided evidence for its role in these processes. PGRPLC silencing increased *P. berghei* infection intensity 4.4-fold (P<0.001) and increased *P. falciparum* infection intensity (P<0.005) and prevalence (from 41% to 52%, P<0.005). PGRPLC also controlled gut microbiota proliferation, with a 2-fold increase in bacterial load following silencing. Structural modelling showed that PGRPLC3 is the most relevant isoform and is capable of binding both DAP-type and Lys-type peptidoglycan, though it cannot initiate dimerization. The study highlighted that PGRPLC-mediated defense influences *Plasmodium* infection, supporting the tripartite interaction between microbiota, immunity, and parasites.

##### 3.5.4.2 PGRPLA and PGRPLB

Gendrin et al. (63) studied the effect of PGRPLA and PGRPLB on *Anopheles* immunity and *Plasmodium* infection. Silencing of PGRPLA1 resulted in enhanced *P. berghei* infection, and silencing of PGRPLA2 resulted in enhanced *P. falciparum* infection. The Imd pathway was positively regulated by PGRPLA1, whose knockdown led to a decrease in the expression of CEC1. The silencing of PGRPLB resulted in an upregulation of CEC1 expression in the fat body and had a significant effect on the decrease in *P. falciparum* infection prevalence, highlighting the negative regulation of the Imd pathway by PGRPLB. These results point to the multiple regulatory controls of mosquito immunity by PGRPs.

##### 3.5.4.3 PGRP-LD and peritrophic matrix

Song et al. (65) demonstrated that PGRP-LD in *An. stephensi* protects the vector from *P. berghei* infection by regulating gut homeostasis. PGRP-LD knockdown (dsLD) increased susceptibility to *P. berghei* and decreased gut microbiota abundance. This dysbiosis led to peritrophic matrix (PM) fragmentation due to dysregulation of PM genes. Antibiotic-treated mosquitoes lost PM integrity, and recolonization with *Enterobacter* sp. restored PM integrity and reduced vector competence. PGRP-LD silencing in antibiotic-treated mosquitoes (without PM) did not increase susceptibility, confirming that the effect is mediated through the PM rather than direct immune action.

##### 3.5.4.4 LRIM1 knockout

Inbar et al. (67) used CRISPR-Cas9 to generate an LRIM1 knockout line (Δaslrim1) in *An. stephensi* to improve *P. falciparum* sporozoite yields for vaccine production. On a surprising note, the bacterial load in Δaslrim1 mosquitoes was 21-fold higher, and the microbiome composition changed significantly as well, with acetic acid bacteria (*Asaia*) being eliminated. This caused increased mortality and, contrary to expectations, significantly reduced *P. falciparum* infection in non-aseptic conditions. LRIM1 deletion was also found to have an impact on reproductive capacity, as oviposition, fecundity, and male fertility were significantly reduced. In this study, we show that LRIM1’s regulation of the microbiome plays a significant role in vector competence and that full knockout of a gene can lead to unexpected phenotypes because of disruption of the microbiome.

##### 3.5.4.5 Bacteria-independent defenses

Blumberg et al. (57) identified bacteria- and IMD pathway-independent anti-*P. falciparum* defenses. Transcriptomic analysis of septic and aseptic *P. falciparum*-infected *An. gambiae* identified a serine protease inhibitor (SRPN7) and a Clip-domain serine protease (CLIPC2) that were transcriptionally induced in aseptic midguts upon infection. SRPN7 silencing decreased *P. falciparum* infection intensity (P < 0.001), indicating that SRPN7 is a negative regulator of anti-*Plasmodium* immunity, while CLIPC2 silencing had no significant effect. Co-silencing assays suggested that SRPN7 and CLIPC2 may function together in a signaling cascade. Neither gene was regulated by *P. berghei* infection, suggesting *P. falciparum*-specific responses. Neither gene regulated the IMD pathway (no effect on TEP1, FBN9, or LRRD7 expression), confirming the existence of bacteria-independent and IMD pathway-independent anti-*Plasmodium* defenses.

##### 3.5.4.6 Microbiota and basal immunity

Dong et al. (55) showed that the mosquito gut microbiota is necessary for inducing basal immunity that limits *Plasmodium* infection. Antibiotic-treated (aseptic) mosquitoes were significantly more susceptible to *P. falciparum* infection (P < 0.01), with a 2.5-fold increase in ookinetes in the midgut epithelium. The immune genes (Cec3, Gambicin, PGRP-LB, LRRD7, TEP1, Rel2) were depleted, causing a significant increase in the abundance of midgut bacteria. The anti-*Plasmodium* effect of some immune genes (Cec3, PGRP-LB, ClipA9) was modulated by the presence of microbiota, suggesting complex interactions between immunity, microbiota, and parasites.

**Table 7** summarises the key immune genes and pathways associated with anti-plasmodium defense.

**Table 7:** Immune genes and pathways involved in anti-*Plasmodium* defense.

Gene/Pathway	Function	Effect on *Plasmodium*	Dependency
PGRPLC	Peptidoglycan recognition; IMD pathway activation	↓ Infection (4.4× increase for *P. berghei*; *P. falciparum* also increased but no fold change reported)	Microbiota-dependent
PGRPLA1	Positive regulator of Imd pathway	↓ Infection	Partially bacteria-dependent
PGRPLA2	Positive regulator of Imd pathway	↓ *P. falciparum* infection	Not specified
PGRPLB	Negative regulator of Imd pathway	Negative regulator of Imd pathway	Gut-mediated
PGRP-LD	Gut homeostasis; PM integrity	↓ Infection via PM	Microbiota-dependent
LRIM1	Complement-like cascade; TEP1 stabilization	↓ Infection (KO increased bacterial load, *reduced P. falciparum*)	Microbiota-mediated
SRPN7	Serine protease inhibitor	↓ Infection (silencing ↓ infection)	Bacteria-independent
CLIPC2	Clip-domain serine protease	Not significant alone	Bacteria-independent
TEP4, TEP15	Thioester-containing proteins	↓ Infection (*Wolbachia*-regulated)	*Wolbachia*-induced
CLIPB2, CLIPB4	Clip-domain serine proteases	↓ Infection (*Wolbachia*-regulated)	*Wolbachia*-induced
Lysozyme C2	Antimicrobial peptide	↓ Infection (*Wolbachia*-regulated)	*Wolbachia*-induced

↓ means decrease, while ↑ means increase.

#### 3.5.5 Microbiota composition and vector competence

Boissiere et al. (18) studied the midgut microbiota of *An. gambiae* from Cameroon and looked for associations with *P. falciparum* infection, using a 16S rRNA pyrosequencing approach. The microbiota was dominated by Proteobacteria (94%) and showed significant inter-individual variability. The abundance of *Enterobacteriaceae* was significantly correlated with *P. falciparum* infection status (P = 0.004), indicating that mosquitoes with Enterobacteriaceae were more likely to be infected. The microbiota composition was influenced by the breeding site where mosquitoes originated, indicating environmental acquisition of bacteria.

Sandeu et al. (79) studied the diversity of microbiota in *An. gambiae* collected from Cameroon and its association with natural infection with *P. falciparum*. Ninety-nine bacterial taxa were identified, of which 97 were common to infected and uninfected mosquitoes. There were two genera unique to uninfected mosquitoes (*Acetobacteraceae* and *Enterococcus*), and none unique to infected mosquitoes. There were 14 genera of bacteria that were more abundant in uninfected mosquitoes, and only 2 more abundant in infected mosquitoes. Significant differences in bacterial composition were observed between infection status (PERMANOVA, q = 0.04) and seasons (PERMANOVA, q = 0.001). Alpha diversity was higher in uninfected samples during the wet season and in infected samples during the dry season.

Bassene et al. (66) performed a comparison study of the microbiota of *P. falciparum-infected* and uninfected *An. gambiae* and *An. funestus* in Senegal. The microbiota was more diverse in infected samples, although total reads were higher in uninfected mosquitoes. Proteobacteria were the most common phylum, and the most common genus in uninfected vectors was *Burkholderia*. Specific bacteria (*Asaia bogorensis*, *Enterobacter cloacae*, *Burkholderia fungorum*, *B. cepacia*) were only detected in the *P. falciparum-negative* samples. In this study, *Wolbachia* was also detected for the first time in Senegal in *An. funestus*.

Tatsinkou Maffo et al. (77) determined the quantities of *Asaia* in field-caught *An. gambiae* and *An. coluzzii* from Cameroon. *Asaia* prevalence was 88.3% in *An. gambiae* and 80.9% in *An. coluzzii*, with seasonal variation in density. There was no correlation between *Asaia* loads and *P. falciparum* infection. Interestingly, in *An. coluzzii*, the density of *Asaia* was higher when the mosquitoes were infected with *Plasmodium* (P < 0.0001), while in *An. gambiae*, the density of *Asaia* was higher when the mosquitoes were not infected with *Plasmodium* (P < 0.0001), indicating species-specific interactions.

**Table 8** summarises the key findings on microbiota composition and its association with P. falciparum infection.

**Table 8:** Microbiota composition and association with *P. falciparum* infection.

Study	Location	Mosquito species	Key finding
Boissière et al. (18)	Cameroon	*An. gambiae*	*Enterobacteriaceae* positively correlated with *P. falciparum* infection.
Bassene et al. (66)	Senegal	*An. gambiae*, *An. funestus*	*Burkholderia* abundant in *P. falciparum*-free samples; microbiota more diverse in infected.
Sandeu et al. (79)	Cameroon	*An. gambiae*	14 genera more abundant in uninfected; 2 in infected; seasonal variation.
Tatsinkou Maffo et al. (77)	Cameroon	*An. gambiae*, *An. coluzzii*	No correlation between *Asaia* and *P. falciparum*; species-specific patterns.

#### 3.5.6 Environmental factors and vector competence

##### 3.5.6.1 Circadian rhythm

The study by Habtewold et al. (69) studied the effect of the circadian rhythm on *P. falciparum* infection in *An. coluzzii*. Parasite gametocytes were significantly more infectious at nighttime than during the day (ZT18 vs ZT7, P < 0.0001). However, mosquito infections were significantly lower when they were reared under a reversed light-dark regime (DL) versus standard light-dark (LD) (P < 0.0001). CLOCK gene silencing reversed infection outcomes: LD mosquitoes showed reduced oocyst load when CLOCK was silenced (P = 0.005), while DL mosquitoes showed increased oocyst load (P = 0.003). Midgut microbiota load was significantly lower at ZT18 (midnight) compared to ZT0 and ZT6 (P < 0.0001), coinciding with elevated lysozyme C-1 expression. The study found that interactions between the mosquito vector, the malaria parasite, and gut microbiota are finely balanced to facilitate the transmission of malaria, and laboratory infection protocols should mimic natural interactions.

##### 3.5.6.2 Artificial light

Llergo et al. (72) tested the effect of artificial light at night on *P. falciparum* infectivity in *An. stephensi*. Photophase significantly reduced median oocyst intensity in both septic (80% reduction) and aseptic (51% reduction) mosquitoes compared to scotophase. Antibiotic treatment increased oocyst numbers, consistent with microbiota-mediated protection. No significant difference was found between the photophase and scotophase for sporozoite production. The study suggests that artificial light could be considered an additional mosquito-control strategy.

##### 3.5.6.3 Seasonality and geography

Several studies reported seasonal variation in microbiota composition and infection prevalence. Sandeu et al. (79) found significant differences in microbiota composition between dry and wet seasons (PERMANOVA, q = 0.001), with higher abundance of certain bacteria in different seasons. Herren et al. (36) reported that *Microsporidia MB* prevalence fluctuated seasonally, with the highest infection rates 4–6 weeks after peak rainfall. Ahouandjinou et al. (74) reported higher *Microsporidia* prevalence in *An. coluzzii* (57%) than *An. gambiae* (41%), suggesting species-specific differences. Ecological niche modelling by Sovi et al. (83) identified elevation (78%) and soil (20%) as the main environmental drivers of *Microsporidia MB* habitat suitability in Benin.

#### 3.5.7 Antibiotics and microbiota disruption

Gendrin et al. (59) studied the influence of penicillin/streptomycin (PS) in ingested human blood on mosquito microbiota and malaria transmission. The bacterial growth in the blood was decreased by 70% with PS supplementation, while the composition of the microbiota was changed (a decrease of 92-fold in Enterobacteriaceae and a 19-fold increase in Acetobacteraceae). Exposure to PS was associated with a 21% increase in *P. falciparum* infection prevalence and a doubling of median oocyst intensity (P = 0.041). Mosquito survival (P = 0.0016) and fecundity (P = 0.0038) also increased. This was confirmed by field experiments with naturally infected blood from children in Burkina Faso that showed higher intensity of infection (P = 0.02). The findings of this study indicated that antibiotic use in malaria endemic regions could be a risk factor for malaria transmission.

Gendrin et al. (60) studied the differential effects of azithromycin, doxycycline, and co-trimoxazole on malaria transmission. Azithromycin resulted in a significant reduction in the intensity of *P. falciparum* infection (42%, P = 5 × 10^-7^) and in the mosquito lifespan (P = 0.037). Doxycycline showed no significant effect at therapeutic concentration but increased infection at high concentration (P = 0.027). Co-trimoxazole had a negative effect on the infection intensity (27% reduction, P = 0.026) but increased prevalence in some experiments. The study showed that the effect of antibiotics on the transmission of malaria is drug-specific, with azithromycin observed to have a clear negative effect on transmission.

#### 3.5.8 Enterobacter and chromobacterium with anti-plasmodium activity

##### 3.5.8.1 *Enterobacter* Esp_Z (ROS-mediated inhibition)

Cirimotich et al. (21) isolated *Enterobacter* sp. Esp_Z from wild *An. arabiensis* populations in Zambia. Esp_Z rendered mosquitoes ~99% resistant to *P. falciparum* infection by interfering with parasite development before midgut epithelium invasion. The anti-*Plasmodium* mechanism was independent of the IMD pathway, as RNAi depletion of PGRP-LC, Imd, and REL2 did not rescue parasite development. Esp_Z inhibited ookinete formation by 98% (both *in vitro* and *in vivo*). The inhibition was dose-dependent and required replicating bacteria, as heat-inactivated bacteria were less effective. Reactive oxygen species (ROS) production by Esp_Z was identified as the mechanism, as vitamin C rescued parasite development in a dose-dependent manner. This study was the first to demonstrate that bacterial ROS production mediates natural refractoriness to *Plasmodium*.

##### 3.5.8.2 *Enterobacter cloacae* ST95 vs ST73

Tchioffo et al. (62) characterized *E. coli* clones isolated from *An. gambiae* midguts and investigated their effects on *P. falciparum* development. Two clones, 444_ST95 and 351_ST73, were compared. *E. coli* 444_ST95 (ST95 complex) significantly reduced *P. falciparum* prevalence (OR 2.47, 95% CI 1.42-4.31, P = 0.001) and intensity (diff mean 1.11, P = 0.01), while 351_ST73 showed no significant effect. The ST95 strain contained the hemolysin F virulence gene (hlyF), which may be involved in virulence. TEM showed that 351_ST73 induced chromatin clumping (which suggests colibactin-induced DNA damage), and 444_ST95 induced vacuolated cytoplasm and loss of microvilli. Differential expression of antimicrobial peptides (cecropin, defensin, gambicin) was observed between the two strains. The results of this study have revealed intra-specific variation in the ability of the bacteria to inhibit *Plasmodium*, and the need for strain-level characterization.

##### 3.5.8.3 Chromobacterium Csp_P

Ramirez et al. (58) isolated *Chromobacterium* sp. Csp_P from field-caught *Ae. aegypti* and tested its activity against *P. falciparum* in *An. gambiae*. Csp_P effectively colonized the mosquito midgut and significantly reduced *P. falciparum* infection. Csp_P also exhibited entomopathogenic activity against both larval and adult stages, with high mortality observed in Csp_P-exposed mosquitoes. *In vitro* experiments demonstrated that Csp_P biofilm, but not planktonic cultures, had anti-*Plasmodium* activity against asexual, ookinete, and gametocyte stages. Csp_P also showed anti-dengue virus activity, suggesting broad-spectrum anti-pathogen properties. The study highlighted the potential of Csp_P as a dual-action agent (mosquito killing + pathogen blocking).

### 3.6 Combined strategies (transgenesis + paratransgenesis)

Huang et al. (68) used a paratransgenesis approach to complement the transgenesis strategy for improving malaria transmission blocking. Transgenic *An. stephensi* expressing anti-*Plasmodium* effectors (scorpine and MP2 peptide) in the midgut and salivary glands were generated using the Q-system for gene expression. Engineered *Serratia* AS1-multi bacteria secreting the same effectors were used to make a transgenic organism (paratransgenesis). Transgenic mosquitoes reduced the number of *P. falciparum* oocysts by 81% and sporozoites by 85%. The reduction in oocysts by paratransgenesis was 69.6%, and for sporozoites it was 65.4% in wild-type mosquitoes. Importantly, transgenesis together with paratransgenesis resulted in the highest level of inhibition: mouse models showed 93% (14/15 mice protected from infection) transmission blocking. This study revealed that combined interventions can complement each other to achieve better transmission blocking and be a powerful tool for malaria control as summarised in **Table 9**. The population of the engineered *Serratia* AS1 was transmitted vertically and horizontally for several generations, which is indicative of field applicability.

**Table 9:** Summary of anti-*Plasmodium* strategies and their efficacy.

Strategy	Effector(s)	Oocyst reduction	Sporozoite reduction	Transmission blocking
Transgenesis (Q-system)	Scorpine + MP2	81%	85%	Not directly assessed
Paratransgenesis	*Serratia* AS1-multi	69.6%	65.4%	Partial
Transgenesis + Paratransgenesis	Scorpine + MP2 (*Serratia*)	>80%	>85%	93% (mouse model)
Engineered *Pantoea*	Scorpine, EPIP	~98%	Not assessed	84% prevalence reduction
*Serratia* Su_YN1 (natural)	AmLip	~98%	Not assessed	High
*Enterobacter* Esp_Z (natural)	ROS	~98%	~99%	High

## 4 Discussion

The data in this systematic review were collected from 41 studies testing the effect of microbiota on the development of the sporozoite of *Plasmodium falciparum* in the vectors, the *Anopheles* mosquitoes. The results indicated that mosquito microbiota play a role in modulating malaria parasites’ ability to infect mosquitoes, but the effect is context-specific, with effects varying according to the taxonomic composition of the mosquito microbiota, mosquito species, and ecological context. The most striking observation from this review is the strain- and species-specific nature of the microbiota-mediated effects on the development of *Plasmodium*. *Wolbachia* provides the clearest example of this variability. While wAlbB stably transinfected into *An. stephensi* consistently reduces oocyst and sporozoite loads (45), naturally occurring *Wolbachia* strains show divergent effects: wAnga-Mali negatively correlates with sporozoite infection (64), wAnD shows similar negative associations (82), yet wAnmo in *An. moucheti* exhibits no correlation with *P. falciparum* despite near-fixation prevalence (47). This indicates that in long-standing *Wolbachia*-host relationships, the protective phenotype might be lost, in line with evolutionary theory that vertically transmitted symbionts will be selected to be less virulent and may not have pathogen-blocking properties for an extended period of time.

Another important finding is that microbial inhibition is stage-specific. Several studies have shown that the effects of the microbes are not consistent throughout the *Plasmodium* life cycle. wAnga-Mali further aids in the development of oocysts, but inhibits sporozoites (64); *Microsporidia MB* prevents sporozoite colonization of salivary glands and reduces oocyst establishment (36), and *Enterobacter* Esp_Z inhibits ookinete formation by ROS (21). This stage specificity highlights the following implications for designing interventions: targeting sporozoite development in the salivary glands may be better for transmission blocking than targeting midgut stages only. The included studies describe several mechanisms of the microbiota’s anti-*Plasmodium* effects. Direct mechanisms involve the secretion of anti-parasitic factors, like the lipase AmLip from *Serratia ureilytica* Su_YN1 (33) into parasites through outer membrane vesicles (71) and the production of ROS by *Enterobacter* Esp_Z (21). Indirect mechanisms include activation of the mosquito IMD pathway (via PGRPLC) (54, 63) and maintenance of the peritrophic matrix through microbiota-mediated homeostasis (65). It has also been discovered that there are bacteria-independent defenses (57), further emphasizing the complexity of anti-Plasmodium immunity.

The most mechanistically detailed case is an example of *Serratia ureilytica* Su_YN1 that uses the phosphatidylcholine scavenging pathway to selectively target parasites through the loading of AmLip on OMVs (71). The interaction is very sophisticated; it’s a cross-kingdom interaction where a commensal bacterium evolved to interact with a eukaryotic parasite, only delivering effector molecules to that one. This mechanism could guide the development of new transmission-blocking methods. The evidence analyzed was indicative of the promise of a microbiota-based approach to control malaria. *Microsporidia* MB shows particular promise due to its vertical transmission, lack of fitness costs, and consistent transmission-blocking activity across multiple African countries (36, 74, 75). The association between *Microsporidia* MB and the L1014F *kdr* resistance allele (83) suggests potential synergies with insecticide-based control, where resistant mosquitoes may be more susceptible to symbiont infection. It is important to note that the prevalence figures reported across studies are site-, species-, and season-specific and cannot be pooled into a single definitive prevalence estimate. The variation observed, from 0-9% in Kenya to 41–57% in Benin, reflects genuine ecological and biological heterogeneity rather than methodological error, and reinforces that microbiota-based interventions will require site-specific baseline surveillance before deployment.

There is also great potential for engineered symbionts. Paratransgenesis using *Pantoea agglomerans* expressing scorpine and EPIP achieved ~98% oocyst reduction (35), while combining transgenesis with paratransgenesis using *Serratia* AS1-multi achieved 93% transmission blocking in mouse models (68). These are alternatives to chemical insecticides, especially for outdoor-biting and resistant populations of vectors. There is an important lesson to be learned from the LRIM1 knockout study by Inbar et al. (67). Immune gene knockout led to higher bacterial loads, lower *Plasmodium* infections in non-aseptic conditions, and compromised fecundity, underscoring the need to consider the microbiome when attempting gene knockout. It underscores the importance of considering fitness trade-offs and side effects of any genetic intervention strategy. Similarly, antibiotic use in malaria-endemic areas may inadvertently increase transmission risk (59, 60). Penicillin/streptomycin increased *P. falciparum* infection by 21% and enhanced mosquito survival and fecundity, while doxycycline at high concentrations also increased infection. These findings have implications for mass drug administration programs in malaria-endemic regions.

The durability of these phenotypes under multi-generational and climate-variable conditions remains unresolved. Evidence on generational stability is symbiont-dependent: *wAlbB* remained stable through 34 generations (45), *Serratia* Su_YN1 persisted for three generations (33), whereas w-Anga transmission declined sharply across two generations (10.6% and 16.7%; 78). *Microsporidia MB* is transgenerational but loses approximately 37–39% of infections during development (73). Long-term field persistence has only been demonstrated once, over a five-year interval (64), and near-fixed *Wolbachia* in *An. moucheti* shows no protective phenotype, suggesting that long-established associations may lose blocking capacity (47). Environmental resilience is similarly uncertain: temperature strongly modulates *Microsporidia MB* dissemination (39), prevalence fluctuates seasonally (36), and light and circadian cues alter infection outcomes (69, 72). No included study combined multi-generational tracking with climate-variable exposure, so the durability of these phenotypes under climate change cannot currently be estimated.

These findings have important implications for malaria vector control. *Microsporidia* MB emerges as a strong candidate for field deployment due to its vertical transmission, lack of fitness costs, and consistent transmission-blocking activity across Africa. *Serratia ureilytica* Su_YN1 offers a scalable non-GMO alternative with up to 98% efficacy. The association between *Microsporidia* MB and insecticide resistance alleles suggests potential synergies with resistance management programs. However, the unintended consequences of LRIM1 knockout and the potential for antibiotics to enhance transmission risk caution against intervention strategies that do not consider microbiome-mediated effects. The use of microbiota strategies is most appropriate as an adjunct, rather than a replacement for, current vector control measures. Only a few studies included in this review directly profiled the microbiota community within the salivary glands in the tissue (34, 82), whereas most evaluated the effect of manipulation of gut microbiota on the load of sporozoites in the gut. This is important because there is clear evidence that the microbiota of the midgut plays an indirect role in the development of sporozoites, while there is no direct evidence that salivary gland-resident microbiota modulate the infectivity of sporozoites. The salivary gland microbiome is thus a high-priority area for future research. However, it is critical to note that the available evidence on the use of these in the field is still preliminary, as microbiota-parasite interactions are highly context-dependent, with the study methods varying between studies, few studies investigating this field longitudinally, and almost no characterization of the microbiome in the salivary glands. It is important to note that one of the findings of this review was the lack of direct salivary gland microbiome studies.

## 5 Limitations and strengths

There are some limitations to this systematic review. First, the protocol was not registered with PROSPERO before the extraction of data. Although methodological choices were predetermined to reduce biases, there is potentially a risk of selective reporting and less opportunity for verification of prespecified outcomes (52). Publication bias was not formally tested, as there was significant heterogeneity, and so meta-analysis was not possible (84). There is some risk of publication bias, however, as positive associations are more likely to be published, potentially leading to overestimation of the effectiveness of microbiota-based intervention.

In addition to methodological heterogeneity within individual studies, there was a high degree of methodological variation across all studies, particularly in mosquito species, methods of microbiota manipulation, mode of infection, and outcome measures, that precluded direct comparability and meta-analysis. The heterogeneity is appropriate, but the conclusions from the cross-study analysis are constrained by this heterogeneity, and the results of the individual studies might not be directly transferable. The overall high risk of bias in blinding domains is a significant limitation, and in vector biology studies, blinding is often technically difficult, which could result in overestimation of intervention effect. The studies had a small number of subjects (n < 20 subjects per study) and were not very well designed, so there was not a lot of statistical power, and there was a strong bias towards using short-term assessments, with few studies reporting long-term persistence of microbial effects.

A disconnect exists between laboratory and field studies, as laboratory colonies contain less diverse microbial communities than natural settings. The geographical distribution was heavily concentrated in West Africa (Burkina Faso n=9, Benin n=4) and East Africa (Kenya n=5, Tanzania n=3), with limited data from Southern Africa (Zambia n=1), limiting generalizability. The review’s emphasis on the salivary gland microbiome is constrained by limited availability of studies that directly characterize salivary gland microbial communities; most studies inferred effects from sporozoite load measurements following midgut microbiota manipulation, providing indirect rather than direct evidence.

Despite these limitations, this review has notable strengths. A comprehensive systematic search was conducted across multiple databases (PubMed, Scopus, Google Scholar) with rigorous screening by two independent reviewers. Broad geographical coverage included 15 countries across Africa, Europe, North America, and Asia. Diverse microbial taxa were explored, including bacteria (*Wolbachia*, *Serratia*, *Enterobacter*, *Asaia*, *Pantoea*, *Chromobacterium*, *E. coli*), microsporidian fungi (*Microsporidia* MB), and whole microbial communities. Inclusion of both laboratory and field studies allowed synthesis of mechanistic insights and ecological relevance. Focus on the sporozoite stage addressed a critical gap, as most previous reviews emphasized midgut stages. Risk of bias assessment using SYRCLE’s tool provided transparency in evaluating methodological quality.

## 6 Future research directions

Long-term stability and transmission of promising candidates of microorganisms like *Microsporidia* MB or *Serratia* Su_YN1 across different ecological settings should be determined through longitudinal field studies in the future. Multi-generational studies conducted under variable temperature, humidity, and photoperiod regimes are urgently needed to determine whether microbiota-mediated blocking phenotypes persist under climate change. Mechanistic research is required to properly understand microbiota-mediated inhibition of the sporozoite, both in the uptake of the OMV by the phosphatidylcholine scavenging pathway and in the immune signaling pathway triggered by the different taxa of bacteria. Standardized protocols for microbiota manipulation and outcome assessment would facilitate meta-analysis and cross-study comparison. The unexpected finding of *kdr* resistance allele association with *Microsporidia* MB prevalence warrants further investigation to determine whether insecticide resistance mechanisms influence symbiont susceptibility or vice versa. Further work is needed to understand the potential to deploy the naturally occurring *Plasmodium*-blocking microbes in the field, covering formulation, delivery methods (such as attractive toxic sugar baits and larval breeding site inoculation), and persistence in the environment. Lastly, there is a lack of research on the salivary gland microbiome, and further studies are needed to describe its composition, its dynamics, and its function in the development and spread of sporozoites.

## 7 Conclusion

The present systematic review brings together the comprehensive evidence indicating that the mosquito microbiota has a significant impact on the development of *Plasmodium falciparum* sporozoites in the vectors *Anopheles*, whose impact is highly strain-, species-, and context-dependent. The transmission-blocking potential of *Wolbachia* is variable, ranging from strong inhibition to no correlation, while the transmission-blocking potential of *Microsporidia* MB is consistent across multiple countries in Africa, and there are no apparent fitness costs. *Serratia ureilytica* Su_YN1 and its AmLip-OMV delivery system are the most mechanistically well-understood natural symbiont, which can be used as a model for generating new transmission-blocking strategies. Integrated approaches are the most promising, with the highest experimental transmission blocking (93%) from transgenesis and paratransgenesis. The LRIM1 knockout study is a reminder, however, that immune gene manipulation may have unintended consequences via microbiome disruption. The results presented here support the development of microbiota-based approaches as sustainable, environmentally friendly alternatives and/or supplements to chemical insecticides, especially for managing populations of vectors such as outdoor-biting and insecticide-resistant ones. Future studies should focus on field validation, mechanistic elucidation, and practical deployment strategies to translate these promising findings into effective malaria control tools.

## Data Availability

The original contributions presented in the study are included in the article/supplementary material. Further inquiries can be directed to the corresponding authors.
